# Physical Activity, Screen Time, Cardiorespiratory Fitness, and Selective Attention in Primary School Children: A Cross-Sectional Study

**DOI:** 10.3390/jfmk11040380

**Published:** 2026-09-23

**Authors:** María del Carmen Carcelén-Fraile, Amanda Camacho-Illana, Víctor López-Cantero, Noemí García-Padilla

**Affiliations:** 1 Department of Educational Sciences, Faculty of Social Sciences, University of Atlántico Medio, 35017 Las Palmas de Gran Canaria, Spain; 2 Department of Didactics of Musical, Plastic and Corporal Expression, University of Jaén, 23071 Jaén, Spain; aci00013@red.ujaen.es (A.C.-I.); victorloc@gmail.com (V.L.-C.); ngp00027@red.ujaen.es (N.G.-P.)

**Keywords:** physical activity, screen time, cardiorespiratory fitness, selective attention, children

## Abstract

**Background:** Physical activity, screen time, and cardiorespiratory fitness have been associated with cognitive functioning in children; however, their mutually adjusted associations with specific dimensions of selective attention remain insufficiently understood. This study examined the associations of habitual physical activity, screen time, and cardiorespiratory fitness with selective attention in primary school children. **Methods:** This cross-sectional study included 386 children aged 9–12 years (54.4% girls). Habitual physical activity was assessed using the Physical Activity Questionnaire for Children (PAQ-C), screen time using the Youth Leisure-Time Sedentary Behavior Questionnaire (YLSBQ), cardiorespiratory fitness using the 20 m shuttle run test, and selective attention using the d2-R Test of Attention. Selective attention was characterized by processing speed (TR), percentage of errors (E%), and concentration performance (CON). Associations were examined using correlation and multiple linear regression analyses adjusted for age and sex, followed by hierarchical regression models examining the mutually adjusted associations of physical activity, screen time, and cardiorespiratory fitness with selective-attention outcomes. **Results:** Higher physical activity was significantly associated with a greater number of total items processed (TR; B = 69.87, β = 0.660, *p* < 0.001), a lower percentage of errors (E%; B = −1.75, β = −0.356, *p* < 0.001), and better concentration performance (CON; B = 26.44, β = 0.659, *p* < 0.001). Greater screen time was primarily associated with a higher error percentage (β = 0.597, *p* < 0.001), while associations with processing speed and concentration performance were weaker. Higher cardiorespiratory fitness was associated with greater processing speed (β = 0.468), fewer errors (β = −0.455), and better concentration performance (β = 0.667; all *p* < 0.001). In the final hierarchical models, after mutual adjustment for physical activity, screen time, cardiorespiratory fitness, age, and sex, physical activity was associated with processing speed (β = 0.600, *p* < 0.001), whereas screen time and cardiorespiratory fitness were not associated with this outcome. For attentional errors, higher screen time (β = 0.523, *p* < 0.001), lower physical activity (β = −0.117, *p* = 0.014), and lower cardiorespiratory fitness (β = −0.270, *p* < 0.001) were associated with a higher error percentage. For concentration performance, both physical activity (β = 0.395, *p* < 0.001) and cardiorespiratory fitness (β = 0.412, *p* < 0.001) were positively associated, whereas screen time was not associated with this outcome. **Conclusions:** Physical activity, screen time, and cardiorespiratory fitness showed distinct associations with specific components of selective attention. Higher physical activity was particularly associated with faster processing speed, higher screen time with greater attentional errors, and both physical activity and cardiorespiratory fitness with better concentration. Given the cross-sectional design, these findings should be interpreted as associations rather than causal relationships.

## 1. Introduction

Childhood is a critical developmental period during which cognitive functions undergo substantial maturation and progressively support increasingly complex academic and everyday demands [1]. Among these functions, selective attention plays a central role in children’s ability to identify task-relevant information while suppressing competing or distracting stimuli [2]. This capacity is particularly relevant in school settings, where learning continuously requires children to sustain goal-directed behavior, process visual information efficiently, and inhibit irrelevant responses [3]. Consequently, identifying modifiable lifestyle factors associated with selective attention has become an important research priority, particularly given the profound changes occurring in children’s daily movement and screen-use patterns [4].

Physical activity has emerged as one of the lifestyle behaviors most consistently associated with cognitive health during childhood [5]. Beyond its well-established physical and psychosocial benefits, accumulating evidence suggests that greater engagement in physical activity may support cognitive domains such as attention and executive functioning [6]. Meta-analytic and systematic-review evidence indicates that physical activity interventions can improve cognitive performance in children, although effects vary according to the cognitive outcome and characteristics of the activity performed [7,8]. Several mechanisms have been proposed, including changes in cerebral blood flow, neurotrophic signaling, neural efficiency, and brain structural and functional development. However, physical activity represents only one of several lifestyle-related factors associated with cognitive functioning [9]. Cardiorespiratory fitness, which is closely related to habitual moderate-to-vigorous physical activity, may therefore also be relevant when examining these relationships [10].

Cardiorespiratory fitness reflects the integrated ability of the cardiovascular and respiratory systems to supply oxygen during sustained physical activity and has been increasingly associated with cognitive functioning in children and adolescents [11]. Previous research has reported positive associations between cardiorespiratory fitness and attention-related outcomes, including selective attention. Children and adolescents with higher levels of cardiorespiratory fitness tend to demonstrate better attentional performance and concentration [12,13,14]. These findings suggest that habitual physical activity and cardiorespiratory fitness are closely related factors that may show distinct associations with attentional performance [15]. However, relatively few studies have simultaneously examined habitual physical activity, cardiorespiratory fitness, and distinct components of selective-attention performance within the same analytical framework [16]. At the same time, children’s daily routines are increasingly characterized by substantial exposure to screen-based behaviors. Screen time is a particularly relevant component of sedentary behavior because of its high prevalence and potential implications for cognitive development [17]. However, it should not be considered synonymous with sedentary behavior, as its cognitive correlates may depend on duration, content, timing, and type of engagement [18]. Systematic reviews have reported that greater screen exposure is frequently associated with poorer attentional outcomes in children, although findings are not unequivocal and longitudinal evidence suggests that these relationships may be bidirectional [19,20]. Screen time may also affect cognitive functioning indirectly by displacing behaviors beneficial for cognitive and physical development, including physical activity, consistent with the broader 24 h movement-behavior framework [21].

Cardiorespiratory fitness may also be relevant when considering the relationship between screen time and attentional functioning. Greater screen exposure may coexist with lower engagement in physically demanding activities and lower cardiorespiratory fitness, whereas higher fitness levels have repeatedly been associated with more favorable cognitive and attentional outcomes [22]. Cardiorespiratory fitness may therefore represent an additional physiological correlate of attentional functioning that is related to, but distinct from, habitual movement behaviors. Yet most previous research has examined physical activity, screen time, fitness, and cognition separately or focused on global cognitive outcomes [23]. Comparatively little attention has been paid to the associations of physical activity, screen time, and cardiorespiratory fitness with specific dimensions of selective attention in primary school children. Moreover, a single global attention score may obscure meaningful differences between processing speed, accuracy, and concentration [24]. This distinction is relevant because selective attention is not a unitary performance characteristic, but rather comprises complementary dimensions of attentional functioning. These include processing speed and visual scanning efficiency, response accuracy, and the ability to select relevant information while inhibiting inappropriate or distracting responses [25]. Importantly, these components may develop differently and contribute independently to attentional performance, with recent evidence showing age-related differences in processing speed, response accuracy, and stimulus discrimination during childhood [26]. Examining these dimensions separately may therefore provide a more nuanced understanding of how children’s movement behaviors and cardiorespiratory fitness relate to attentional functioning and whether these associations differ across processing speed, accuracy, and concentration [27]. Such an approach may offer a more precise characterization of the cognitive correlates of physical activity and fitness than relying on a single global indicator of attention [28].

Against this background, the present study aimed to examine the associations of habitual physical activity, screen time, and cardiorespiratory fitness with different dimensions of selective attention in primary school children and to determine whether these factors were mutually adjusted associations with attentional outcomes after accounting for age and sex. Based on previous evidence, we hypothesized that higher physical activity and cardiorespiratory fitness would be associated with better selective-attention performance, whereas higher screen time would be associated with poorer performance. We further hypothesized that physical activity, screen time, and cardiorespiratory fitness would show distinct patterns of association with specific selective-attention outcomes in mutually adjusted models.

## 2. Methods

### 2.1. Study Design and Participants

An observational, analytical, cross-sectional study was conducted in a sample of primary school children aged 9–12 years. Participants were recruited from primary schools that agreed to take part in the study using a non-probability convenience sampling strategy. Of the 395 children initially approached, 386 were ultimately included in the study, corresponding to a response rate of 97.72%. The final sample comprised 176 boys (45.6%) and 210 girls (54.4%).

Children were eligible for participation if they met the following criteria: (i) being enrolled in primary education, (ii) being between 9 and 12 years of age, and (iii) having written informed consent provided by a parent or legal guardian. Children were excluded when they presented a medical condition or physical limitation that could interfere with their ability to participate in physical activity assessments. Participants with incomplete or missing data for the main study variables were also excluded from the analyses.

An a priori sample size calculation was performed using GPower version 3.1. Because the primary objective was to examine associations between movement-related variables and selective attention outcomes, the initial sample size calculation was based on the detection of a small bivariate association as a conservative criterion. Assuming a two-tailed correlation, an alpha level of 0.05, a statistical power of 80%, and a small expected effect size (r = 0.15), a minimum sample of 340 participants was required. To account for an anticipated 10% of incomplete questionnaires or invalid data, the target sample size was increased to 374 participants. In addition, sample-size adequacy for the hierarchical regression analyses was evaluated using the “Linear multiple regression: Fixed model, R^2^ increase” procedure in GPower. Considering the incremental association of cardiorespiratory fitness after accounting for age, sex, physical activity, and screen time (one tested predictor and five predictors in the final model), an alpha level of 0.05, statistical power of 80%, and a small incremental effect size of f^2^ = 0.021, a minimum sample of 376 participants was required. The final sample of 386 children therefore exceeded the sample size required for both the bivariate and hierarchical regression analyses (Figure 1).

### 2.2. Measures

#### 2.2.1. Selective Attention

Selective attention was assessed using the Spanish adaptation of the d2-R Test of Attention—Revised [29], adapted by Ruiz-Fernández and published by Hogrefe TEA Ediciones. The d2-R is a timed cancellation test consisting of 14 rows of 47 stimuli (658 items in total). Each stimulus consists of the letter “d” or “p” accompanied by one to four marks positioned above and/or below the letter. Participants were instructed to identify and cross out only the letter “d” accompanied by exactly two marks, regardless of their position, while ignoring all other stimuli. A maximum of 20 s was allowed for each row. Three indices were considered in the present study. Total items processed (TR) represents the total number of stimuli processed within the allotted time, with higher scores indicating greater visual processing speed. Percentage of errors (E%) represents the proportion of omission and commission errors relative to the total number of items processed, with lower scores indicating greater accuracy. Concentration performance (CON) reflects the number of correctly identified target stimuli while accounting for commission errors, with higher scores indicating better concentration and selective attention performance. The d2-R has demonstrated high internal consistency for its main indices in previous studies, with Cronbach’s α coefficients ranging from 0.90 to 0.97. In the present sample, the instrument showed good internal consistency (Cronbach’s α = 0.836).

#### 2.2.2. Physical Activity

Habitual physical activity was assessed using the Physical Activity Questionnaire for Children (PAQ-C) [30], a self-administered questionnaire designed to evaluate general physical activity levels in school-aged children during the previous seven days. The PAQ-C assesses participation in moderate-to-vigorous physical activity across different contexts, including physical education classes, recess, lunchtime, after school, evenings, and weekends. The questionnaire comprises nine items used to derive the overall physical activity score, each rated on a 5-point scale. The final PAQ-C score is calculated as the mean of the individual item scores and ranges from 1 to 5, with higher scores indicating higher levels of habitual physical activity. The PAQ-C is intended to provide an overall estimate of habitual physical activity rather than the frequency, duration, or intensity of specific activities. The Spanish version of the PAQ-C has demonstrated satisfactory psychometric properties in school-aged children, including good internal consistency (Cronbach’s α = 0.83). In the present sample, the PAQ-C showed acceptable internal consistency (Cronbach’s α = 0.710).

#### 2.2.3. Screen Time

Screen time was assessed using the Youth Leisure-Time Sedentary Behavior Questionnaire (YLSBQ), a self-reported instrument designed to assess sedentary behaviors performed during leisure time in children and adolescents [31]. The questionnaire covers several domains of sedentary behavior, including screen-based and non-screen-based activities. For the purposes of the present study, screen-based behaviors were specifically considered, including time spent watching television, playing video games, using a computer, and using a mobile phone. Participants reported the time spent in each screen-based activity separately for school days and weekend days. Following the YLSBQ scoring procedure, responses were converted into time values, and the average daily duration of each screen-based behavior was calculated as [(school-day time × 5) + (weekend-day time × 2)]/7. Total daily screen time was subsequently calculated by summing the weighted daily estimates for television viewing, video game use, computer use, and mobile phone use, and was expressed in hours per day, with higher values indicating greater daily screen exposure. No additional adjustment was applied for the simultaneous use of multiple screen devices; therefore, total screen time represents the summed reported exposure across the four screen-based behaviors rather than mutually exclusive clock time. The YLSBQ has demonstrated adequate psychometric properties for the assessment of sedentary behaviors in young populations, including satisfactory reliability and internal consistency. In the present sample, the screen-time items showed acceptable internal consistency (Cronbach’s α = 0.791).

#### 2.2.4. Cardiorespiratory Fitness

Cardiorespiratory fitness was assessed using the 20 m shuttle run test (Course Navette), a progressive field-based test widely used to evaluate aerobic fitness in children and adolescents [32]. Participants were required to run continuously back and forth between two lines positioned 20 m apart, following the pace dictated by standardized audio signals. The test started at a speed of 8.5 km/h, which increased by 0.5 km/h every minute. Participants were instructed to maintain the required pace for as long as possible. The test was terminated when the participant was unable to reach the 20 m line in time with the audio signal on two consecutive occasions or stopped because of volitional exhaustion. Performance was recorded according to the final stage reached, with higher values indicating greater cardiorespiratory fitness. The 20 m shuttle run test has demonstrated good criterion-related validity and high test–retest reliability for the assessment of cardiorespiratory fitness in children and adolescents.

### 2.3. Procedure

Prior to data collection, the study protocol was presented to the management teams of the participating primary schools, and institutional authorization was obtained. Written informed consent was subsequently obtained from the parents or legal guardians of all participating children. Each participant was assigned a unique identification code to ensure anonymity and confidentiality throughout data collection and analysis.

Data collection was conducted during regular school hours in scheduled sessions coordinated with the teaching staff. Assessments were carried out in designated areas within the schools under standardized conditions. Members of the research team provided standardized instructions before each assessment and remained present throughout the sessions to clarify any questions and ensure that participants correctly understood the procedures. Teachers were also present to facilitate organization and maintain an appropriate assessment environment.

Habitual physical activity and screen-based sedentary behavior were assessed using the PAQ-C and YLSBQ, respectively. Questionnaires were administered in the classroom under researcher supervision. Participants completed the questionnaires individually after receiving standardized instructions, and researchers were available to resolve any doubts without influencing their responses. Selective attention was assessed using the d2-R Test of Attention under standardized classroom conditions. Instructions were provided according to the standardized administration protocol, and participants completed each of the 14 rows within the established 20 s time limit per row. Cardiorespiratory fitness was assessed using the 20 m shuttle run test in an appropriate school facility. Participants followed the standardized audio-paced protocol and were encouraged to maintain the required running pace for as long as possible. The test was terminated according to the predefined criteria described above, and the final stage reached was recorded. All assessments were conducted following standardized procedures to minimize measurement variability and ensure consistency across participants and participating schools.

### 2.4. Statistical Analysis

Statistical analyses were performed using IBM SPSS Statistics version 22.0 (IBM Corp., Armonk, NY, USA). Continuous variables were summarized as means and standard deviations (SD), whereas categorical variables were expressed as frequencies and percentages. Prior to inferential analyses, the distribution of continuous variables and the presence of potential outliers were examined.

Bivariate associations between physical activity, screen time, cardiorespiratory fitness, and selective attention were examined using Pearson’s correlation coefficients. Selective attention was evaluated using three indicators derived from the d2-R Test of Attention: total number of items processed (TR), percentage of errors (E%), and concentration performance (CON). Higher TR and CON scores reflected better attentional performance, whereas higher E% values indicated lower accuracy.

Multiple linear regression analyses were performed to examine the associations of physical activity and screen time with each selective-attention outcome (TR, E%, and CON). Separate models were fitted for physical activity and screen time, with age and sex included as covariates. Unstandardized regression coefficients (B), standardized coefficients (β), 95% confidence intervals (CI), and coefficients of determination (R^2^) were reported. Given the availability of covariates, these regression models were minimally adjusted for age and sex. The assumptions of linear regression, including linearity, normality and homoscedasticity of residuals, independence of errors, and absence of problematic multicollinearity, were assessed.

Finally, hierarchical multiple linear regression analyses were performed separately for TR, E%, and CON to examine the mutually adjusted associations of physical activity, screen time, and cardiorespiratory fitness with selective-attention outcomes. Age and sex were entered in Block 1 as covariates. Physical activity and screen time were entered simultaneously in Block 2, followed by cardiorespiratory fitness in Block 3. Changes in explained variance (ΔR^2^) were examined at each step and interpreted as the increase in the proportion of variance explained by the specified model after the addition of each block of predictors. Interpretation of the hierarchical analyses focused primarily on the regression coefficients from the final models, in which physical activity, screen time, cardiorespiratory fitness, age, and sex were included simultaneously. Collinearity was assessed using tolerance and variance inflation factor (VIF) values.

Given the correlation between physical activity and cardiorespiratory fitness, a sensitivity analysis was also performed to examine the dependence of block-specific ΔR^2^ values on predictor entry order. The hierarchical analyses were repeated with age and sex entered in Block 1, cardiorespiratory fitness and screen time entered simultaneously in Block 2, and physical activity entered in Block 3. The sensitivity analysis was used to assess the robustness of the interpretation of block-specific changes in explained variance rather than to alter the specification of the final regression models.

All statistical tests were two-tailed, and statistical significance was set at *p* < 0.05.

Given the cross-sectional design of the study, regression coefficients and changes in explained variance were interpreted as associations and not as evidence of temporal, causal, or mediational relationships.

## 3. Results

### 3.1. Participant Characteristics

A total of 386 primary school children were included in the analyses, comprising 176 boys (45.6%) and 210 girls (54.4%). Participants were aged between 9 and 12 years, with a mean age of 11.15 ± 0.66 years. Overall, 280 children (72.5%) reported participating in extracurricular sports, with a mean weekly sport participation of 3.12 ± 2.35 h. The mean physical activity score was 2.88 ± 0.65, while average screen time was 2.77 ± 0.93 h per day. Mean cardiorespiratory fitness was 5.70 ± 2.29. Regarding selective attention, mean TR, E%, and CON scores were 395.42 ± 68.43, 8.43 ± 3.17%, and 152.81 ± 25.93, respectively. Participant characteristics are summarized in Table 1.

### 3.2. Bivariate Associations

Pearson’s correlation analyses showed that physical activity was positively associated with cardiorespiratory fitness (r = 0.620, *p* < 0.001), total items processed (TR; r = 0.653, *p* < 0.001), and concentration performance (CON; r = 0.650, *p* < 0.001), and negatively associated with the percentage of errors (E%; r = −0.352, *p* < 0.001).

Screen time showed a different pattern of associations. Higher screen time was associated with lower cardiorespiratory fitness (r = −0.214, *p* < 0.001), lower TR scores (r = −0.105, *p* = 0.040), lower CON scores (r = −0.188, *p* < 0.001), and a higher percentage of errors (r = 0.597, *p* < 0.001). Physical activity and screen time were weakly and inversely correlated (r = −0.131, *p* = 0.010).

Cardiorespiratory fitness was positively associated with TR (r = 0.468, *p* < 0.001) and CON (r = 0.669, *p* < 0.001), and negatively associated with E% (r = −0.455, *p* < 0.001). Overall, higher levels of physical activity and cardiorespiratory fitness were associated with better selective attention performance, whereas greater screen time was associated with a less favorable attentional profile (Table 2).

### 3.3. Associations Between Physical Activity and Selective Attention

Multiple linear regression analyses were performed to examine the associations between physical activity and selective attention outcomes after adjustment for age and sex (Table 3). Higher physical activity was significantly associated with a greater number of total items processed (TR; B = 69.87, 95% CI: 61.82 to 77.93; β = 0.660; *p* < 0.001). The model explained 43.3% of the variance in TR (adjusted R^2^ = 0.428; F(3, 382) = 97.19, *p* < 0.001). Higher physical activity was also associated with a lower percentage of errors (E%; B = −1.75, 95% CI: −2.21 to −1.29; β = −0.356; *p* < 0.001). This model explained 12.6% of the variance in E% (adjusted R^2^ = 0.120; F(3, 382) = 18.44, *p* < 0.001). Finally, physical activity was positively associated with concentration performance (CON; B = 26.44, 95% CI: 23.39 to 29.48; β = 0.659; *p* < 0.001), with the model explaining 43.5% of the variance in CON (adjusted R^2^ = 0.431; F(3, 382) = 98.16, *p* < 0.001). Overall, higher physical activity levels were consistently associated with better selective attention performance across all three d2-R indicators, independently of age and sex.

### 3.4. Associations Between Physical Screen Time and Selective Attention

Multiple linear regression analyses were performed to examine the association between total daily screen time and selective attention outcomes, adjusting for age and sex. For total performance (TR), the overall regression model was not statistically significant, F(3, 382) = 1.518, *p* = 0.209, explaining 1.2% of the variance (R^2^ = 0.012; adjusted R^2^ = 0.004). Nevertheless, total screen time showed a small but statistically significant negative association with TR (B = −7.72, β = −0.104, 95% CI [−15.11, −0.33], *p* = 0.041). Neither age nor sex was significantly associated with TR.

In contrast, the model predicting error percentage was statistically significant, F(3, 382) = 70.536, *p* < 0.001, and explained 35.6% of the variance (R^2^ = 0.356; adjusted R^2^ = 0.351). Greater screen time was strongly associated with a higher percentage of errors (B = 2.05, β = 0.597, 95% CI [1.77, 2.32], *p* < 0.001), whereas age and sex were not significantly associated with error percentage. The model predicting concentration performance was also statistically significant, F(3, 382) = 5.244, *p* = 0.001, although it explained a relatively small proportion of the variance (R^2^ = 0.040; adjusted R^2^ = 0.032). Greater screen time was independently associated with lower concentration performance (B = −5.24, β = −0.187, 95% CI [−8.00, −2.48], *p* < 0.001) (Table 4). Again, neither age nor sex showed significant associations with concentration performance.

Overall, greater screen time showed differential associations across selective attention outcomes. The strongest association was observed for error percentage, whereas the association with concentration performance was modest and that with processing speed was weak.

### 3.5. Cardiorespiratory Fitness and Selective Attention

Multiple linear regression analyses were performed to examine the associations between cardiorespiratory fitness and selective attention outcomes, adjusting for age and sex (Table 5). Higher cardiorespiratory fitness was significantly associated with a greater total number of items processed (TR; B = 13.99, 95% CI: 11.33 to 16.64, β = 0.468, *p* < 0.001) and better concentration performance (CON; B = 7.56, 95% CI: 6.71 to 8.41, β = 0.667, *p* < 0.001). Conversely, higher cardiorespiratory fitness was associated with a lower percentage of errors (E%; B = −0.63, 95% CI: −0.76 to −0.51, β = −0.455, *p* < 0.001). The models explained 22.0% of the variance in TR, 20.8% of the variance in E%, and 44.9% of the variance in CON. Age and sex were not significantly associated with any of the selective attention outcomes in these models.

### 3.6. Mutually Adjusted Associations of Physical Activity, Screen Time, and Cardiorespiratory Fitness with Selective Attention

Hierarchical multiple regression analyses were performed to examine the mutually adjusted associations of physical activity, screen time, and cardiorespiratory fitness with each selective-attention outcome after adjustment for age and sex (Table 6).

For TR, the covariate-only model including age and sex was not significant (R^2^ = 0.001, adjusted R^2^ = −0.004, *p* = 0.844). The addition of physical activity and screen time in Block 2 increased the explained variance by 43.2% (ΔR^2^ = 0.432, *p* < 0.001). Adding cardiorespiratory fitness in Block 3 produced only a small and non-significant increase in explained variance (ΔR^2^ = 0.005, *p* = 0.063), resulting in a final R^2^ of 0.438 (adjusted R^2^ = 0.431). In the final model, physical activity remained strongly associated with TR (β = 0.600, *p* < 0.001), whereas screen time (β = −0.005, *p* = 0.897) and cardiorespiratory fitness (β = 0.093, *p* = 0.063) were not independently associated with TR.

For E%, age and sex explained virtually no variance (R^2^ = 0.001, adjusted R^2^ = −0.005, *p* = 0.906). The addition of physical activity and screen time in Block 2 increased the explained variance by 43.3% (ΔR^2^ = 0.433, *p* < 0.001). The subsequent addition of cardiorespiratory fitness further increased the explained variance by 4.3% (ΔR^2^ = 0.043, *p* < 0.001), yielding a final R^2^ of 0.477 (adjusted R^2^ = 0.470). In the final model, higher screen time was independently associated with a higher percentage of errors (β = 0.523, *p* < 0.001), whereas higher physical activity (β = −0.117, *p* = 0.014) and higher cardiorespiratory fitness (β = −0.270, *p* < 0.001) were independently associated with a lower percentage of errors.

For CON, the covariate-only model was not significant (R^2^ = 0.005, adjusted R^2^ = −0.001, *p* = 0.421). The addition of physical activity and screen time in Block 2 increased the explained variance by 44.1% (ΔR^2^ = 0.441, *p* < 0.001). Adding cardiorespiratory fitness in Block 3 produced a further 10.1% increase in explained variance (ΔR^2^ = 0.101, *p* < 0.001), resulting in a final R^2^ of 0.546 (adjusted R^2^ = 0.540). In the final model, both physical activity (β = 0.395, *p* < 0.001) and cardiorespiratory fitness (β = 0.412, *p* < 0.001) were independently associated with higher concentration performance, whereas screen time was not independently associated with CON (β = −0.047, *p* = 0.185).

Collinearity diagnostics for the final models showed tolerance values ranging from 0.593 to 0.994 and VIF values ranging from 1.007 to 1.687, indicating no evidence of problematic multicollinearity among the predictors.

In the sensitivity analyses reversing the order of entry of physical activity and cardiorespiratory fitness, the block-specific changes in explained variance differed from those observed in the primary hierarchical analyses. For TR, adding cardiorespiratory fitness and screen time in Block 2 increased the proportion of variance explained by 21.9% (ΔR^2^ = 0.219, *p* < 0.001), followed by a further 21.9% after adding physical activity in Block 3 (ΔR^2^ = 0.219, *p* < 0.001). For E%, the corresponding changes were 46.8% (ΔR^2^ = 0.468, *p* < 0.001) and 0.8% (ΔR^2^ = 0.008, *p* = 0.014), respectively. For CON, they were 44.7% (ΔR^2^ = 0.447, *p* < 0.001) and 9.4% (ΔR^2^ = 0.094, *p* < 0.001), respectively. The final R^2^ values remained unchanged (TR: R^2^ = 0.438; E%: R^2^ = 0.477; CON: R^2^ = 0.546), because the final models contained the same set of predictors irrespective of entry order. These findings indicate that block-specific ΔR^2^ values were dependent on predictor entry order and should therefore not be interpreted as measures of the relative importance of individual predictors.

## 4. Discussion

The present study examined the associations of habitual physical activity, screen time, and cardiorespiratory fitness with different dimensions of selective attention in primary school children. The main findings indicate that higher levels of physical activity and cardiorespiratory fitness were consistently associated with a more favorable attentional profile, whereas greater screen exposure was associated with poorer selective attention performance. Importantly, the associations differed according to the specific attentional outcome examined. In the final mutually adjusted models, physical activity was associated with processing speed, screen time was associated with response accuracy, and both physical activity and cardiorespiratory fitness were associated with concentration performance. These findings suggest that movement behaviors and physical fitness may be related to distinct, although complementary, dimensions of selective attention during childhood.

### 4.1. Physical Activity and Selective Attention

Higher habitual physical activity was associated with better performance across all three d2-R outcomes after adjustment for age and sex. Children reporting higher physical activity processed a greater number of stimuli, committed a lower percentage of errors, and achieved higher concentration performance. The magnitude of these associations was particularly notable for processing speed (TR) and concentration performance (CON), with the corresponding models explaining approximately 43% of the variance in both outcomes.

These findings are consistent with previous evidence supporting a positive relationship between physical activity and cognitive functioning in children. Meta-analytic evidence has shown that physical activity is associated with improvements in several cognitive domains during childhood, including attentional and executive processes. More specifically, interventions incorporating cognitively engaging physical activity have demonstrated beneficial effects on executive functioning, with particularly relevant improvements in inhibitory control [8]. Similarly, previous meta-analytic evidence in children aged 4–12 years suggests that physical activities incorporating cognitive demands may enhance executive functioning [33].

Several mechanisms may help explain these associations. Regular physical activity has been linked to neurobiological adaptations relevant to cognitive functioning, including enhanced cerebral oxygenation and neurotransmission, as well as structural and functional adaptations in brain regions and networks involved in executive control [34]. Beyond these physiological mechanisms, the cognitive demands inherent to many forms of physical activity may also be relevant. Activities requiring continuous monitoring of environmental information, rapid decision-making, response inhibition, and adaptation to changing situations repeatedly engage cognitive control processes. In this regard, recent meta-analytic evidence indicates that cognitively engaging physical activity can improve executive functioning in children and adolescents, particularly inhibitory control [8], while the magnitude of cognitive benefits may vary according to the type of physical activity performed. Thus, the favorable attentional profile observed among more active children in the present study may reflect both physiological adaptations associated with regular physical activity and the repeated cognitive demands embedded within physically active behaviors.

In the final mutually adjusted model, habitual physical activity was associated with processing speed, whereas cardiorespiratory fitness was not associated with this outcome after accounting for age, sex, screen time, and physical activity. However, the sensitivity analysis showed that the block-specific changes in explained variance depended on the order in which physical activity and cardiorespiratory fitness were entered into the hierarchical model. When physical activity and screen time were entered before cardiorespiratory fitness, the corresponding ΔR^2^ values were 0.432 and 0.005, respectively, whereas reversing the order resulted in ΔR^2^ values of 0.219 and 0.219. Therefore, these sequential changes in explained variance should not be interpreted as evidence of greater relative importance of physical activity compared with cardiorespiratory fitness. Rather, interpretation should focus on the final mutually adjusted model, which was unchanged by predictor entry order. Given the correlation between physical activity and cardiorespiratory fitness, these findings are consistent with shared explained variance between the two constructs. Previous evidence suggests that the cognitive associations of physical activity may vary according to exercise modality and the cognitive demands involved [8,33]. However, because the PAQ-C does not characterize the specific cognitive or coordinative demands of the activities performed, the present study cannot determine whether these characteristics contributed to the observed association with processing speed. Further studies are needed to clarify the respective relationships of physical activity and cardiorespiratory fitness with processing speed.

### 4.2. Screen Time and Selective Attention

Screen exposure showed a different pattern of associations with selective attention. Greater daily screen time showed differential associations across the three d2-R indicators, with substantial variation in the magnitude of these relationships. The association with error percentage (E%) was β = 0.597, with the adjusted model explaining 35.6% of its variance. In contrast, the associations with processing speed (TR; β = −0.104) and concentration performance (CON; β = −0.187) were considerably smaller. These findings are broadly consistent with previous evidence linking greater screen exposure with poorer attentional outcomes in children. A systematic review by Santos et al. [19] found that most of the included studies reported associations between greater screen time and attention problems in typically developing children. Similarly, a subsequent systematic review reported consistent associations between higher screen exposure and attentional difficulties across cross-sectional studies, although findings from longitudinal studies were less uniform [20].

The particularly strong association with E% suggests that screen exposure may be more closely related to the accuracy of attentional performance than to processing speed itself. Children with greater screen exposure did not process substantially fewer stimuli, but they tended to commit more errors during the task. This distinction is potentially relevant because attention is not a unitary construct, and screen exposure may relate differently to the speed and accuracy components of attentional performance. Previous research has also linked adherence to lower recreational screen-time recommendations with favorable cognitive outcomes. For example, in a large sample of 8–11-year-old children from the Adolescent Brain Cognitive Development study, meeting the recreational screen-time recommendation was associated with better global cognition [35]. However, the mechanisms underlying the particularly strong association with error rate observed in the present study remain uncertain and warrant further investigation.

One possible explanation is that the characteristics of digital media exposure, not only its duration, may be relevant to attentional functioning. Different forms of screen use vary considerably in their content, pace, interactivity, and cognitive demands, and total screen-time measures cannot capture these qualitative differences. Consequently, the present findings should not be interpreted as indicating that all forms of screen exposure have equivalent associations with attention. Moreover, evidence regarding the temporal direction of this relationship remains inconclusive. A systematic review of attentional outcomes in young children found evidence suggesting that the association may be bidirectional: greater screen exposure may precede attentional difficulties, but children with poorer attentional regulation may also be more likely to engage with screen-based media [35]. Given the cross-sectional design of the present study, no temporal or causal direction can be established.

Screen time was also only weakly inversely correlated with physical activity in the present sample (r = −0.131). This finding suggests that screen-based sedentary behavior and physical activity should not necessarily be viewed as opposite ends of a single behavioral continuum. Children may accumulate relatively high levels of physical activity while simultaneously engaging in substantial screen use. This interpretation is consistent with the 24 h movement behavior framework, in which physical activity, sedentary behavior, and sleep are considered distinct but interrelated behaviors whose combination across the day may be relevant to cognitive and other health outcomes. Previous research has shown that adherence to combinations of movement recommendations is associated with more favorable cognitive outcomes in children, supporting the value of considering screen time alongside, rather than simply as the absence of, physical activity [36,37]. Overall, the outcome-specific pattern observed in the present study is noteworthy. Whereas physical activity showed its strongest associations with processing speed and concentration performance, screen exposure was particularly associated with response accuracy. This finding reinforces the importance of examining separate dimensions of selective attention rather than relying exclusively on a global cognitive score. Future studies should determine whether the association with attentional errors is replicated using objective measures of screen exposure and should distinguish between device type, content, purpose, timing, and interactive versus passive screen use.

### 4.3. Cardiorespiratory Fitness and Selective Attention

Cardiorespiratory fitness was consistently associated with selective attention performance. After adjustment for age and sex, higher fitness was associated with greater processing speed and concentration performance and with a lower percentage of errors. The association with concentration performance was β = 0.667, with the model explaining 44.9% of its variance. These findings are consistent with previous research supporting a positive relationship between aerobic fitness and cognitive functioning in children. A systematic review by Van Waelvelde et al. [38] reported generally favorable associations between aerobic fitness and executive functioning in children, although the magnitude and consistency of these relationships varied across cognitive domains and methodological approaches. More recent systematic evidence similarly suggests that higher cardiorespiratory fitness is associated with better performance in cognitive domains including attention, inhibitory control, and working memory.

Particularly relevant to the present findings, Wu et al. [16] examined 1086 children aged 6–12 years using the 20 m shuttle run to assess aerobic fitness and the d2 Attention Test to assess attention. Aerobic fitness was positively associated with attentional performance and was identified as a mediator of the relationship between physical activity and attention. The similarity in age range and assessment methods makes these findings particularly relevant to the present study. However, our results provide a more differentiated perspective by examining processing speed, error rate, and concentration performance separately rather than considering attention as a single outcome.

Several mechanisms may underlie the relationship between cardiorespiratory fitness and cognitive performance. Higher aerobic fitness has been associated with cardiovascular and cerebrovascular adaptations that may support cerebral perfusion, oxygen delivery, and neural efficiency, as well as structural and functional adaptations in brain networks involved in cognitive control. Childhood may represent a particularly relevant developmental period in this regard because of the ongoing maturation and plasticity of neural systems supporting executive and attentional processes. Nevertheless, the precise mechanisms linking cardiorespiratory fitness with cognition remain incompletely understood, and current evidence does not support attributing these associations to a single physiological pathway. Importantly, the hierarchical analyses showed that the mutually adjusted association of cardiorespiratory fitness varied across dimensions of selective attention after accounting for age, sex, physical activity, and screen time. Its addition to the model was not significantly associated with additional explained variance in processing speed (ΔR^2^ = 0.005, *p* = 0.063), whereas it was associated with increases of 4.3% in explained variance for error percentage (ΔR^2^ = 0.043, *p* < 0.001) and 10.1% for concentration performance (ΔR^2^ = 0.101, *p* < 0.001). Thus, cardiorespiratory fitness should not be interpreted as a general correlate uniformly associated with all aspects of attentional performance; rather, its mutually adjusted association varied according to the specific attentional dimension examined.

### 4.4. Mutually Adjusted Associations of Physical Activity, Screen Time, and Cardiorespiratory Fitness

The hierarchical analyses provide further insight into the mutually adjusted associations of habitual physical activity, screen time, and cardiorespiratory fitness with selective attention. The hierarchical analyses provide further insight into the mutually adjusted associations of habitual physical activity, screen time, and cardiorespiratory fitness with selective attention. Different patterns of association were observed across the three attentional outcomes. In the final mutually adjusted model for TR, physical activity was associated with processing speed (β = 0.600, *p* < 0.001), whereas screen time (β = −0.005, *p* = 0.897) and cardiorespiratory fitness (β = 0.093, *p* = 0.063) were not associated with this outcome. For attentional accuracy, higher screen time was associated with a higher percentage of errors (β = 0.523, *p* < 0.001), whereas higher physical activity (β = −0.117, *p* = 0.014) and higher cardiorespiratory fitness (β = −0.270, *p* < 0.001) were associated with fewer errors. For concentration performance, both physical activity (β = 0.395, *p* < 0.001) and cardiorespiratory fitness (β = 0.412, *p* < 0.001) were associated with higher CON scores, whereas screen time was not associated with this outcome (β = −0.047, *p* = 0.185).

These findings can be considered alongside previous research examining relationships among physical activity, aerobic fitness, and cognitive performance. Wu et al. [16], for example, reported that aerobic fitness mediated the association between physical activity and attention in school-aged children. The present study did not formally test mediation and therefore cannot determine whether cardiorespiratory fitness represents an indirect pathway linking physical activity or screen time with selective attention. Instead, our findings show different patterns of mutually adjusted association between physical activity, screen time, cardiorespiratory fitness, and specific components of attentional performance. This outcome-specific pattern further supports considering these variables as related but non-interchangeable correlates of selective attention. Physical activity and cardiorespiratory fitness should therefore not be regarded as interchangeable constructs. Physical activity represents a behavior characterized by its frequency, intensity, duration, type, and context, whereas cardiorespiratory fitness is a physiological attribute influenced by habitual physical activity but also by genetic, maturational, and other individual factors. Longitudinal evidence supports a relationship between physical activity and cardiorespiratory fitness during childhood and adolescence, while also indicating that the two constructs are not equivalent [32]. However, the present study did not assess specific characteristics of physical activity beyond those captured by the PAQ-C, and the cross-sectional design does not allow the mechanisms underlying the observed associations to be established. Whether characteristics such as the type, context, or cognitive and coordinative demands of physical activity are relevant to specific attentional outcomes remains a hypothesis that should be directly investigated in future studies.

More broadly, the previous literature examining physical activity or fitness in isolation has produced heterogeneous findings regarding childhood cognition, and systematic reviews have highlighted variability according to the cognitive domain assessed, fitness measure used, and characteristics of the study population. The present findings suggest that part of this heterogeneity may arise from treating attention as a unitary outcome or considering physical activity and cardiorespiratory fitness separately. Simultaneously examining behavioral activity and physiological fitness, while distinguishing processing speed, accuracy, and concentration performance, may therefore provide a more comprehensive characterization of their relationships with selective attention.

### 4.5. Strengths and Limitations

Several strengths should be considered. The study included a relatively large sample of 386 children, exceeding the minimum sample size estimated a priori. Selective attention was evaluated using a standardized instrument that allowed three distinct components of performance to be examined rather than relying on a single global measure. In addition, physical activity, screen time, and objectively assessed cardiorespiratory fitness were examined simultaneously, and the hierarchical analyses provided information about their associations with selective-attention outcomes beyond age and sex.

Several limitations should also be acknowledged. First, the cross-sectional design precludes establishing temporal or causal relationships. Thus, although physical activity, screen time, and cardiorespiratory fitness were associated with selective attention, it cannot be determined whether these characteristics influence attentional performance or whether children with different cognitive profiles engage differently in these behaviors. Second, physical activity and screen time were assessed using self-reported questionnaires, which may be affected by recall and reporting bias. Objective measures such as accelerometry and device-based screen-use records could provide more precise estimates in future studies. Third, total screen time does not distinguish between different types of devices, content, or purposes of use. Educational screen use, passive viewing, gaming, and social or interactive use may have different relationships with cognitive outcomes. Fourth, the regression models were minimally adjusted for age and sex. Potentially relevant confounding factors such as body composition/adiposity, sleep, socioeconomic characteristics, academic performance, and other lifestyle factors were not collected and therefore could not be included in the regression models or examined in sensitivity analyses. These factors may be associated with both movement behaviors/cardiorespiratory fitness and cognitive performance, and residual confounding therefore cannot be excluded. Accordingly, the mutually adjusted associations observed in the present study should not be interpreted as associations independent of all relevant confounding factors. Future studies should incorporate objective measures of body composition, sleep, and other relevant behavioral and socioeconomic characteristics to examine the robustness of these associations. Finally, although school grade was recorded, class-level identifiers were not retained in the final analytical dataset, which prevented a formal assessment of potential within-class clustering. Therefore, the regression analyses were conducted at the individual level. While the inclusion of students from multiple classes provided a heterogeneous school-based sample, some degree of within-class similarity cannot be excluded. Future studies should retain class-level identifiers to allow the potential clustering of observations to be formally evaluated and, where appropriate, incorporated into the statistical analyses.

### 4.6. Implications and Future Research

Future longitudinal studies should determine whether changes in physical activity, screen exposure, and cardiorespiratory fitness precede changes in selective attention across childhood. Intervention studies would also help establish whether increasing physical activity or improving aerobic fitness produces measurable improvements in specific attentional domains. Furthermore, objectively measured physical activity and sedentary behavior would allow the intensity, duration, and temporal distribution of movement behaviors to be investigated.

Future research should also examine screen exposure in greater detail, considering device type, content, timing, and patterns of use. Given the strong association observed between screen time and error percentage, investigating whether particular forms of screen use are specifically related to inhibitory control or attentional accuracy may be especially relevant.

Finally, the differentiated associations observed for TR, E%, and CON highlight the importance of preserving distinct cognitive outcomes. Rather than treating selective attention as a unitary construct, future studies may benefit from examining processing speed, accuracy, and concentration separately to identify the behavioral and physiological factors most closely related to each component.

## 5. Conclusions

Higher levels of habitual physical activity and cardiorespiratory fitness were associated with a more favorable selective attention profile in primary school children, whereas greater screen time showed less favorable associations that varied across attentional outcomes. Importantly, these associations differed across specific dimensions of selective attention. In the final mutually adjusted models, physical activity was associated with processing speed, screen time was associated with attentional errors, and both physical activity and cardiorespiratory fitness were associated with concentration performance.

These findings highlight that physical activity, screen exposure, and cardiorespiratory fitness showed different patterns of association with attentional functioning during childhood and should not be considered interchangeable constructs. They also emphasize the importance of examining processing speed, accuracy, and concentration separately rather than treating selective attention as a single global outcome. Although the cross-sectional design precludes causal inference, the present results support the relevance of considering physical activity, cardiorespiratory fitness, and screen-use patterns when examining factors associated with children’s cognitive functioning. Longitudinal and intervention studies are needed to determine the temporal and potentially causal relationships underlying these associations.

## Figures and Tables

**Figure 1:**
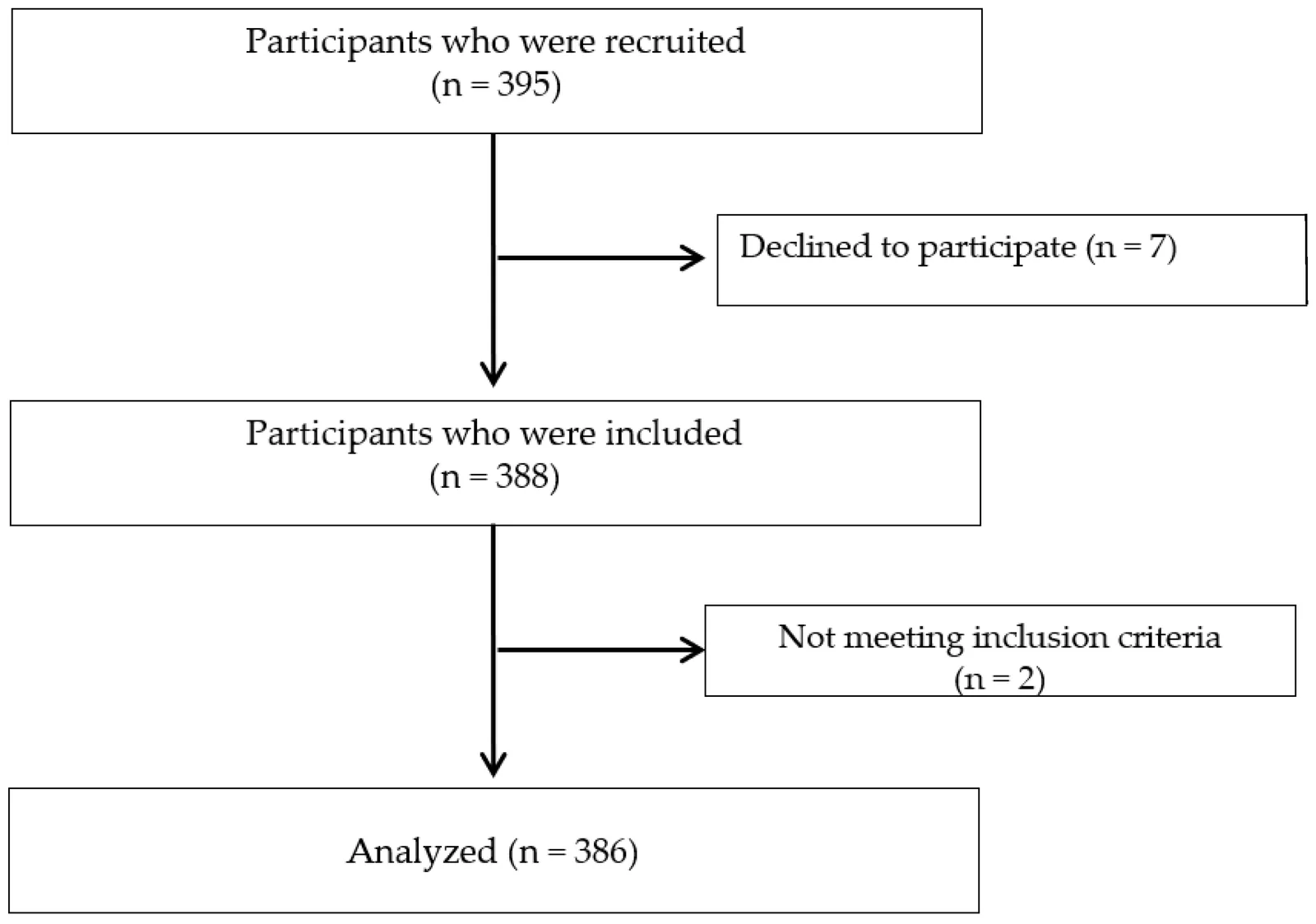
Flowchart of participants.

**Table 1:** Characteristics of the study participants (N = 386).

Variable		Mean ± SD/n (%)
Age (years)		11.15 ± 0.66
Sex	Girl	210 (54.4%)
	Boy	176 (45.6%)
Extracurricular sport participation	Yes	280 (72.5%)
	No	106 (27.5%)
Weekly sport participation (h/week)		3.12 ± 2.35
Physical activity (PAQ-C score)		2.88 ± 0.65
Screen time (h/day)		2.77 ± 0.93
Cardiorespiratory fitness		5.70 ± 2.29
d2-R total items processed (TR)		395.42 ± 68.43
d2-R percentage of errors (E%)		8.43 ± 3.17
d2-R concentration performance (CON)		152.81 ± 25.93

Data are presented as mean ± standard deviation (SD) for continuous variables and n (%) for categorical variables. TR, total number of items processed; E%, percentage of errors; CON, concentration performance; PAQ-C, Physical Activity Questionnaire for Children.

**Table 2:** Pearson correlations between physical activity, screen time, cardiorespiratory fitness, and selective attention outcomes (N = 386).

Variable	1	2	3	4	5	6
1. Physical activity (PAQ-C)	—					
2. Screen time	−0.131 *	—				
3. Cardiorespiratory fitness	0.620 **	−0.214 **	—			
4. TR	0.653 **	−0.105 *	0.468 **	—		
5. E%	−0.352 **	0.597 **	−0.455 **	−0.284 **	—	
6. CON	0.650 **	−0.188 **	0.669 **	0.495 **	−0.501 **	—

Values represent Pearson’s correlation coefficients (r). PAQ-C, Physical Activity Questionnaire for Children; TR, total number of items processed; E%, percentage of errors; CON, concentration performance. Higher E% scores indicate poorer performance. * *p* < 0.05; ** *p* < 0.01.

**Table 3:** Adjusted associations between physical activity and selective attention outcomes (N = 386).

Outcome	B	95% CI for B	β	*p*	R^2^	Adjusted R^2^
TR	69.87	61.82 to 77.93	0.660	<0.001	0.433	0.428
E%	−1.75	−2.21 to −1.29	−0.356	<0.001	0.126	0.120
CON	26.44	23.39 to 29.48	0.659	<0.001	0.435	0.431

B, unstandardized regression coefficient; β, standardized regression coefficient; CI, confidence interval; TR, total number of items processed; E%, percentage of errors; CON, concentration performance. Separate multiple linear regression models were performed for each selective attention outcome. All models were adjusted for age and sex.

**Table 4:** Adjusted associations between screen time and selective attention outcomes (N = 386).

Outcome	B	95% CI for B	β	*p*	R^2^	Adjusted R^2^
TR	−7.72	−15.11 to −0.33	−0.104	0.041	0.012	0.004
E%	2.05	1.77 to 2.32	0.597	<0.001	0.356	0.351
CON	−5.24	−8.00 to −2.48	−0.187	<0.001	0.040	0.032

B, unstandardized regression coefficient; β, standardized regression coefficient; CI, confidence interval; TR, total number of items processed; E%, percentage of errors; CON, concentration performance. Separate multiple linear regression models were performed for each selective attention outcome. All models were adjusted for age and sex.

**Table 5:** Adjusted associations between cardiorespiratory fitness and selective attention outcomes (N = 386).

Outcome	B	95% CI for B	β	*p*	R^2^	Adjusted R^2^
TR	13.99	11.33 to 16.64	0.468	<0.001	0.220	0.213
E%	−0.63	−0.76 to −0.51	−0.455	<0.001	0.208	0.201
CON	7.56	6.71 to 8.41	0.667	<0.001	0.449	0.445

B, unstandardized regression coefficient; β, standardized regression coefficient; CI, confidence interval; TR, total number of items processed; E%, percentage of errors; CON, concentration performance. Separate multiple linear regression models were performed for each selective attention outcome. All models were adjusted for age and sex.

**Table 6:** Hierarchical multiple regression for selective attention outcomes (N = 386).

Outcome/Model	Predictor	B	SE	95% CI for B	β	*p*	Tolerance	VIF	R^2^	Adjusted R^2^	ΔR^2^
TR											
Model 1	Age	2.32	5.30	—	0.022	0.661			0.001	−0.004	—
	Sex	−2.44	7.03	—	−0.018	0.729					
Model 2	Age	2.49	4.00	—	0.024	0.533			0.433	0.427	0.432 ***
	Sex	−9.82	5.33	—	−0.072	0.066					
	Physical activity	69.63	4.14	—	0.657	<0.001					
	Screen time	−1.29	2.88	—	−0.018	0.653					
Model 3	Age	2.61	3.99	[−5.23, 10.45]	0.025	0.513	0.994	1.007	0.438	0.431	0.005
	Sex	−8.90	5.33	[−19.39, 1.59]	−0.065	0.096	0.978	1.023			
	Physical activity	63.62	5.23	[53.34, 73.90]	0.600	<0.001	0.607	1.649			
	Screen time	−0.38	2.91	[−6.10, 5.34]	−0.005	0.897	0.954	1.048			
	Cardiorespiratory fitness	2.79	1.49	[−0.15, 5.72]	0.093	0.063	0.593	1.687			
E%				—							
Model 1	Age	0.01	0.25	—	0.001	0.978			0.001	−0.005	—
	Sex	0.14	0.33	—	0.023	0.657					
Model 2	Age	−0.00	0.19	—	−0.001	0.979			0.434	0.428	0.433 ***
	Sex	0.24	0.25	—	0.038	0.330					
	Physical activity	−1.38	0.19	—	−0.282	<0.001					
	Screen time	1.92	0.13	[−0.37, 0.33]	0.559	<0.001					
Model 3	Age	−0.02	0.18	[−0.35, 0.59]	−0.004	0.908	0.994	1.007	0.477	0.470	0.043 ***
	Sex	0.12	0.24	[−1.04, −0.12]	0.018	0.627	0.978	1.023			
	Physical activity	−0.58	0.23	[1.54, 2.05]	−0.117	0.014	0.607	1.649			
	Screen time	1.79	0.13	[−0.51, −0.24]	0.523	<0.001	0.954	1.048			
	Cardiorespiratory fitness	−0.37	0.07	—	−0.270	<0.001	0.593	1.687			
CON				—							
Model 1	Age	−1.84	2.00	—	−0.047	0.359			0.005	−0.001	—
	Sex	−2.70	2.66	—	−0.052	0.311					
Model 2	Age	−1.77	1.50	—	−0.045	0.240			0.446	0.440	0.441 ***
	Sex	−5.38	2.00	—	−0.103	0.007					
	Physical activity	25.89	1.55	[−4.24, 1.10]	0.645	<0.001					
	Screen time	−2.85	1.08	[−7.40, −0.26]	−0.102	0.008					
Model 3	Age	−1.57	1.36	[12.34, 19.35]	−0.040	0.249	0.994	1.007	0.546	0.540	0.101 ***
	Sex	−3.83	1.82	[−3.27, 0.63]	−0.074	0.036	0.978	1.023			
	Physical activity	15.84	1.78	[3.66, 5.66]	0.395	<0.001	0.607	1.649			
	Screen time	−1.32	0.99		−0.047	0.185	0.954	1.048			
	Cardiorespiratory fitness	4.66	0.51		0.412	<0.001	0.593	1.687			

TR = total number of items processed; E% = percentage of errors; CON = concentration performance; B = unstandardized regression coefficient; SE = standard error; β = standardized regression coefficient; R^2^ = coefficient of determination; ΔR^2^ = change in explained variance. Model 1 included age and sex; Model 2 additionally included physical activity and screen time; Model 3 additionally included cardiorespiratory fitness. *** *p* < 0.001 for ΔR^2^.

## Data Availability

The data presented in this study are not publicly available due to privacy and ethical restrictions related to the participation of minors. Data may be available from the corresponding author upon reasonable request.

## Abbreviations

The following abbreviations are used in this manuscript:

PAQ-C	Physical Activity Questionnaire for Children
YLSBQ	Youth Leisure-Time Sedentary Behavior Questionnaire
TR	Total number of items processed
E%	Percentage of errors
CON	Concentration performance
PA	Physical activity
CRF	Cardiorespiratory fitness
SD	Standard deviation
CI	Confidence interval
B	Unstandardized regression coefficient
β	Standardized regression coefficient
R^2^	Coefficient of determination
ΔR^2^	Change in explained variance
r	Pearson’s correlation coefficient
